# Bioactive fatty acid analog-derived hybrid nanoparticles confer antibody-independent chemo-immunotherapy against carcinoma

**DOI:** 10.1186/s12951-023-01950-y

**Published:** 2023-06-08

**Authors:** Xi Tan, Chenhui Wang, Hong Zhou, Shuting Zhang, Xuhan Liu, Xiangliang Yang, Wei Liu

**Affiliations:** 1 College of Life Science and Technology, Huazhong University of Science and Technology, Wuhan, 430074 P.R. China; 2 The Key Laboratory for Human Disease Gene Study of Sichuan Province, Department of Laboratory Medicine, Sichuan Provincial People’s Hospital, University of Electronic Science and Technology of China, Chengdu, 611731 P.R. China; 3 Department of Emergency Medicine, Shenzhen University General Hospital, Shenzhen University Clinical Medical Academy, Shenzhen, 518060 P.R. China; 4 National Engineering Research Center for Nanomedicine, Huazhong University of Science and Technology, Wuhan, 430074 P.R. China

**Keywords:** Polymer-lipid hybrid nanoparticles, Helper-polymer, 2-Bromopalmitate, PD-L1, Chemo-immunotherapy

## Abstract

Typical chemo-immunotherapy against malignant carcinoma, is characterized by the combined application of chemotherapeutic agents and monoclonal antibodies for immune checkpoint blockade (ICB). Temporary ICB with antibodies would not depress tumor intrinsic PD-L1 expression and potential PD-L1 adaptive upregulation during chemotherapy, thus exerting limited immunotherapy efficacy. Herein, we developed novel polymer-lipid hybrid nanoparticles (2-BP/CPT-PLNs) for inducing PD-L1 degradation by inhibiting palmitoylation with bioactive palmitic acid analog 2-bromopalmitate (2-BP) to replace PD-L1 antibody (*α*PD-L1) for ICB therapy, thus achieving highly efficient antitumor immune via immunogenic cell death (ICD) induced by potentiated chemotherapy. GSH-responsive and biodegradable polymer-prodrug CPT-ss-PAEEP_10_ assisted as a cationic helper polymer could help to stabilize 2-BP/CPT-PLNs co-assembled with 2-BP, and facilitate the tumor site-specific delivery and intracellular release of water-insoluble camptothecin (CPT) in vivo. 2-BP/CPT-PLNs would reinforce cytotoxic CD8^+^ T cell-mediated antitumor immune response via promoting intratumoral lymphocytes cells infiltration and activation. 2-BP/CPT-PLNs significantly prevented melanoma progression and prolonged life survival of mice beyond the conventional combination of irinotecan hydrochloride (CPT-11) and *α*PD-L1. Our work first provided valuable instructions for developing bioactive lipid analogs-derived nanoparticles via lipid metabolism intervention for oncotherapy.

**Graphical Abstract:**

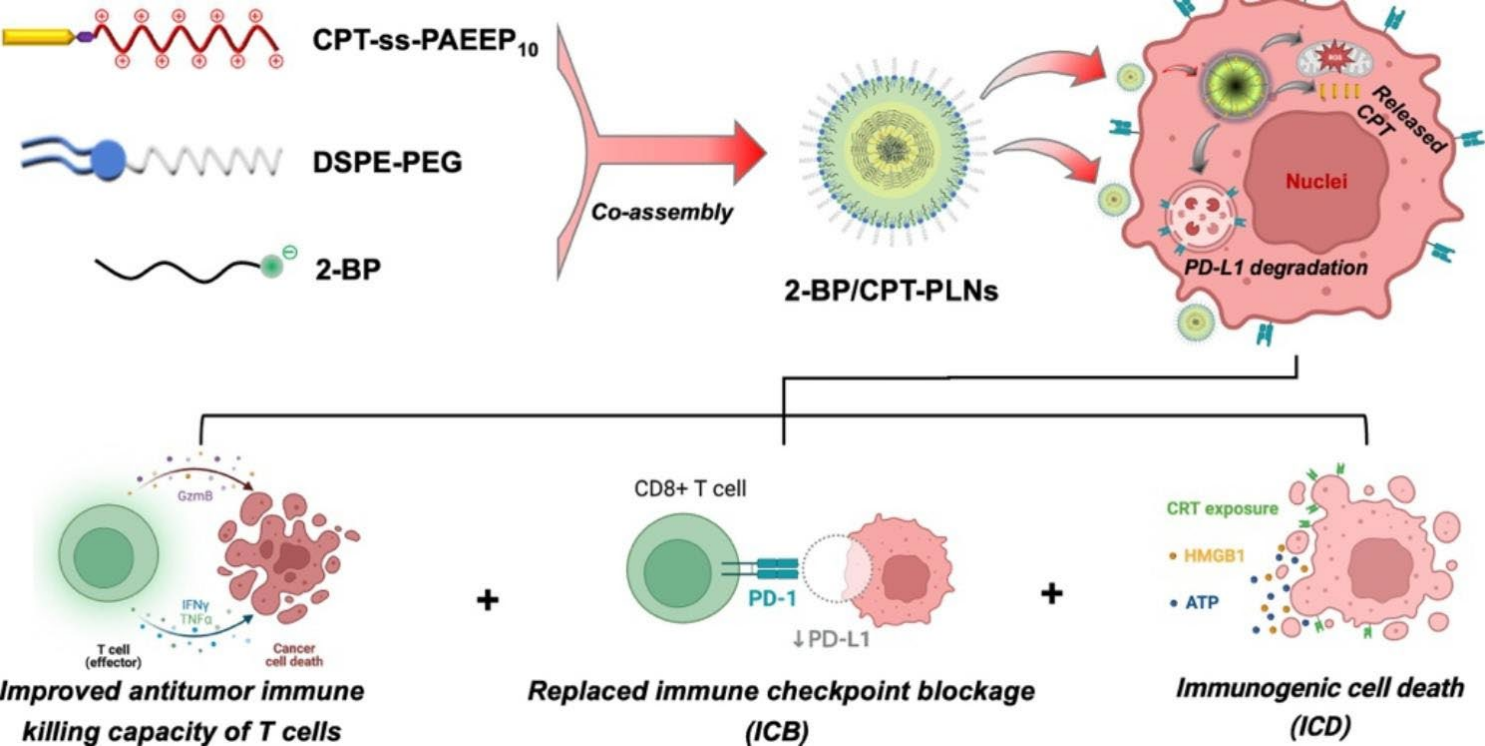

**Supplementary Information:**

The online version contains supplementary material available at 10.1186/s12951-023-01950-y.

## Introduction

Chemo-immunotherapy, one of the most promising antineoplastic combinational therapies, is still under the spotlight of preclinical research and clinical application [1]. Immune checkpoint blockade (ICB) with monoclonal antibodies has been regarded as the gold standard of antitumor immunotherapy [2]. Actually, there is a usually ignored fact that high-expensive monoclonal antibodies such as αPD-L1 temporarily inhibit the interaction of PD-L1 on the extracellular membrane of tumor cells and PD-1 on the surface of T cells, which has no influence on the intrinsic PD-L1 level of carcinoma cells. Downregulating PD-L1 expression of tumors via small molecule therapeutics has been a prospective strategy for evading resistance to conventional monoclonal antibodies [3, 4]. Recently, numerous studies have revealed that palmitoylation of proteins plays a crucial role in stabilizing its membrane anchoring and preventing its ubiquitination for further degradation in lysosomes [5–8]. Palmitoylated inhibitor 2-bromopalmitate (2-BP) as a bioactive fatty acid analog, has been demonstrated to decrease PD-L1 level of extracellular membrane and deplete its storage in recycling endosomes in multiple tumors (breast cancer, colon cancer, bladder cancer, et.), thus promoting T cells immune response for antitumor immunotherapy [9–11]. It was also reported that 2-BP could sensitize adriamycin chemotherapy against osteosarcoma cells and overcome the chemo-resistance of acute promyelocytic leukemia [12]. Therefore, 2-BP as a cheap lipid alternative to replacing expensive αPD-L1 might potentially sensitize chemotherapy to strengthen immunogenic cell death (ICD) for enhanced intratumoral lymphocytes cells infiltration, and promote cytotoxic T cell killing capacity via inducing PD-L1 degradation of cancer cells to achieve the synergistic chemo-immunotherapy [13, 14].

Nevertheless, the poor water solubility and potential lipotoxicity of 2-BP still limit its application for cancer immunotherapy in vivo [15, 16]. Moreover, the dose-dependent limitation of 2-BP as a small molecule inhibitor remains to be solved. Inspired by the conventional solid lipids nanoparticles prepared by palmitic acid (PA), there is a chance to utilize 2-BP as one core component of lipid-based nanoparticles to deliver the chemotherapeutic agents for combined antitumor chemo-immunotherapy [17–21]. Given that 2-BP exhibits stronger electronegativity and polarity from partial ionization because of its lower pKa (~3.0) than PA, there is a logical choice that cationic helper-polymers and stabilizers such as DSPE-PEG are simultaneously introduced to balance the excessive negative charges and hydrophilicity of potential nanoparticles [22, 23]. Polyphosphoesters-based camptothecin prodrug (CPT-ss-PAEEP_n_) was selected as an ideal cationic helper-polymer for preparing the rational hybrid prodrug nanoparticles, owning to its good biosafety, hydrophilicity, rich positive charges, and GSH-responsive drug release according to our previous researches [24–26]. Furthermore, the hydrophobicity/hydrophilicity and drug loading efficiency of CPT-ss-PAEEP_n_ could be flexibly adjusted with the tunable length of polyphosphoesters blocks via controllable synthesis, to facilitate the fabrication of hybrid nanoparticles with 2-BP and DSPE-PEG [27–29].

**Fig. 1:**
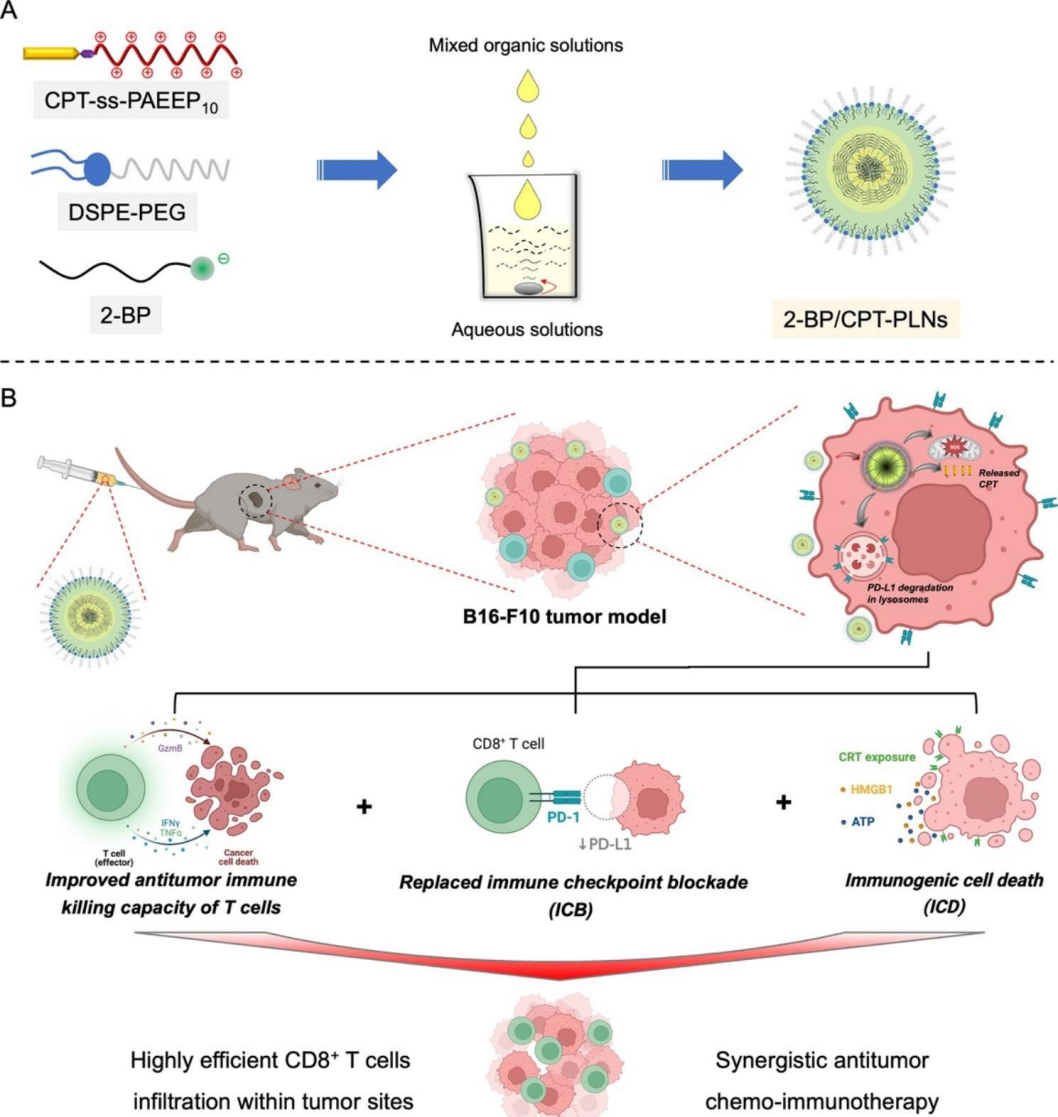
The rational design and synergistic chemo-immunotherapeutic mechanisms of 2-BP/CPT-PLNs. **A** 2-BP/CPT-PLNs were co-assembled from 2-BP, CPT-ss-PAEEP_10_, and DSPE-PEG, and fabricated by the evaporation-emulsification method. **B** Schematic illustration of 2-BP/CPT-PLNs for chemo-immunotherapy via inducing ICD, replacing ICB via inducing PD-L1 degradation, and improving antitumor immune killing capacity of T cells in vivo

Therefore, we developed a novel prodrug-based polymer-lipid hybrid nanoparticles (2-BP/CPT-PLNs) co-assembled from 2-BP, CPT-ss-PAEEP_10_, and DSPE-PEG to achieve the synergistic antitumor chemo-immunotherapy (Fig. 1A). To overcome the tumor adaptive immunotherapy resistance accompanied by upregulation of PD-L1 induced by chemotherapy, it is necessary to promote T lymphocytes cells infiltration and activation, and alleviate the immunosuppressive microenvironment in tumor sites for the highly efficient efficacy of antitumor chemo-immunotherapy [30, 31]. As illustrated in Fig. 1B, 2-BP/CPT-PLNs could efficiently accumulate in tumor sites through blood circulation, boost CPT and 2-BP release with the help of intracellular high-level GSH after their entrance into tumor cells, upregulate cytotoxic CD8^+^ T cells infiltration via reinforcing ICD with CPT chemosensitization, and enable the replaced ICB and improved antitumor immune killing capacity of cytotoxic T cells via targeting PD-L1 degradation and regulating cytokines in tumor sites. Surprisingly, 2-BP/CPT-PLNs significantly prevented melanoma progression and prolonged life survival of mice beyond the conventional combination of irinotecan hydrochloride (CPT-11) and αPD-L1. Thus, our work provides a perspective for designing novel nanomedicine to achieve antibody-independent antitumor chemo-immunotherapy.

## Materials and methods

### Materials and reagents

CPT-ss-PAEEP_10_ was synthesized and characterized according to our previous research [24]. 1,2-stearoyl-sn-glycerol-3-phosphoethanolamine-N-[methoxy(polyethylene glycol)-2000] (DSPE-PEG) was purchased from Shanghai Ponsure Biotech, Inc. 2-BP was purchased from Sigma-Aldrich (St. Louis, MO, USA). Camptothecin (CPT) and irinotecan hydrochloride (CPT-11) were obtained from Aladdin (Shanghai, China). GoInVivo™ purified anti-mouse PD-L1 antibody (αPD-L1, clone: 10F.9G2) was purchased from BioLegend Inc (San Diego, CA, USA). All other antibodies used for flow cytometry or IHC/IF in this study were listed in Table S1 (Additional file 1).

### Preparation and characterization of 2-BP/CPT-PLNs

2-BP/CPT-PLNs were prepared by solvent evaporation and ultrasonic emulsification method as described previously [32]. Typically, CPT-ss-PAEEP_10_, 2-BP, and DSPE-PEG were co-dissolved in methanol-chloroform mixed solvent and then dropwise added to deionized water under stirring. The above mixture was evaporated under heating and stirring overnight, then sonicated under an ice-bath for 2 min. The collected suspension was washed with deionized water by ultrafiltration (Millipore, 10 kDa) to obtain 2-BP/CPT-PLNs after removing the residual organic solvent.

The particle size, polydispersity index (PDI), and zeta potential of 2-BP/CPT-PLNs were measured by dynamic light scattering (DLS) technology (Zetasizer Nano ZS90, Malvern). The size and morphology of nanoparticles were characterized by transmission electron microscopy (TEM, H-7000FA, Hitachi). The encapsulation efficacy of CPT-ss-PAEEP_10_ and 2-BP within 2-BP/CPT-PLNs nanoparticles was measured by fluorescence spectrometer (F-4500, Hitachi) and high-performance liquid chromatography (HPLC, Agilent LC1100, USA), respectively. The assembly behavior, stability, and CPT release behavior of 2-BP/CPT-PLNs were also evaluated by the DLS technology or fluorescence spectrometer.

### In vitro drug release

GSH-responsive drug release of 2-BP/CPT-PLNs was also analyzed by a dialysis method in vitro [33]. Briefly, 2-BP/CPT-PLNs (1 mL) was packaged in a dialysis bag (1000 Da) and then immersed into PBS medium (40 mL, 10 mM, pH 7.4) with or without GSH for further incubation at 37 °C with constant shaking (100 rpm). Subsequently, aliquots (2 mL) were withdrawn from the above PBS medium and replenished with the corresponding fresh dialysate to maintain the constant volumes at predetermined intervals. The CPT contents of the released dialysates were monitored by a multi-mode microplate reader (FlexStation 3, Molecular Devices) with 365 nm excitation and 430 nm emission.

### Cell culture

The murine fibroblast cell line (NIH-3T3), murine breast cancer cell line (4T1) and murine melanoma cell line (B16-F10) were all obtained from the Type Culture Collection of the Chinese Academy of Sciences. All cells were cultured with medium containing fetal bovine serum (10%, Gibco) and penicillin-streptomycin (1%, HyClone), and maintained in a CO_2_ (5%) incubator at 37 °C. Herein, 4T1 cells were grown in RPMI 1640 medium (HyClone) and other cell lines were grown in DMEM medium (HyClone).

### Cellular uptake analysis

B16-F10 cells were seeded in 12-well plates at a density of 2.0 × 10^5^ cells per well and incubated overnight. Then cells were treated with free CPT or 2-BP/CPT-PLNs at an equivalent CPT concentration (10 µg/mL) for a period of time (2 h, 4 h, 6 h). At the predesigned time points, the cells were harvested and resuspended in ice-cold PBS for analysis by flow cytometry (FCM, CytoFLEX S, Beckman). The cellular uptake of 2-BP/CPT-PLNs was further visualized by confocal imaging. Briefly, 1.0 × 10^5^ cells B16-F10 cells were seeded onto confocal dishes overnight and then treated with free CPT or 2-BP/CPT-PLNs as previously described. Subsequently, the cells were fixed with 4% paraformaldehyde (PFA), washed with PBS containing 1% BSA and 0.1% Triton X-100, then stained with Tubulin-Tracker Green and PI (Beyotime) and in darkness. The cells were further imaged with confocal laser scanning microscopy (CLSM) at excitation wavelengths of 405 nm (CPT, blue), 488 nm (tubulin, green) and 640 nm (nuclei, red).

### In vitro cytotoxicity assay

To evaluate the cytotoxicity of 2-BP, CPT, their combination (CPT + 2-BP) and 2-BP/CPT-PLNs, MTT assay was conducted to analyze the cell viability of these cells after the above treatments. Briefly, the cells were seeded into 96-well plates (5 × 10^3^ cells/well) and incubated for cell attachment. Then cells were treated with the above different formulations at a range of concentrations for 48 h. Herein, the molar concentration of 2-BP was fixed at 8-fold that of CPT. After treatments, the cells were incubated with 100 µL MTT (1 mg/mL) in fresh medium for 4 h and then disintegrated with 150 µL DMSO to extract formazan crystals. The resulting 96-well plates were measured by a microplate reader at 570 nm.

### Cell cycle and apoptosis assay

To further explore the synergistic antitumor effects of 2-BP/CPT-PLNs in vitro, we studied their influences on the cell cycle and induced cell apoptosis. Briefly, B16-F10 cells were cultured in 12-well or 6-well plates and conducted by pipette tip to achieve a wound for further incubation with 2-BP, CPT, CPT + 2-BP, and 2-BP/CPT-PLNs for 24 h. The cell cycle arrest and apoptosis induced by 2-BP/CPT-PLNs were analyzed by FCM. The resulting cells were collected, fixed with 70% ethanol (*v/v*), washed with ice-cold PBS, and stained with PI solutions containing RNase A (Beyotime) for FCM measurements to analyze cell cycle ratios. Similarly, the resulting cells were harvested and immediately stained with Annexin V-FITC/PI (Yeasen) to analyze the cell apoptosis by FCM.

B16-F10 multicellular tumor spheroids (MCTs) were also developed to evaluate the cell apoptosis induced by 2-BP/CPT-PLNs as previous report [34]. In brief, B16-F10 cells were seeded onto the U-bottom ultra-low adsorption microplates culture plate at 1000 cells/well to yield B16F10 MCTs. Then these MCTs were treated with CPT, CPT + 2-BP, or 2-BP/CPT-PLNs for 24 h. After removing the old medium containing drugs, the resulting MCTs were immediately stained with Hoechst 33,342 and PI (Beyotime), and imaged with CLSM.

### Evaluation of ICD induced by 2-BP/CPT-PLNs

ICD induced by 2-BP/CPT-PLNs was evaluated by analyzing calreticulin (CRT) exposure, adenosine triphosphate (ATP), and high-mobility group box 1 (HMGB1) release from B16-F10 cells after treatment [35]. Briefly, B16-F10 cells were seeded in 12-well plates (2 × 10^5^ cells/well) and incubated for 24 h. Then the cells were treated with 2-BP, CPT, CPT + 2-BP, and 2-BP/CPT-PLNs at a concentration of 5 µg/mL CPT for 24 h. After treatments, the above cells’ extracellular ATP and HMGB1 concentration were measured with Enhanced ATP Test Kit (Beyotime) or HMGB1 ELISA Test Kit (Solarbio) following the standard protocols. The CRT expression level of the above-treated cells was analyzed by FCM and CLSM after being stained with a primary anti-CRT antibody and Alexa Fluor 488-labeled goat anti-rabbit IgG in turn. Moreover, reactive oxygen species (ROS) production induced by 2-BP/CPT-PLNs was also evaluated with 2’,7’-dichlorofluorescin diacetate (DCFH-DA) fluorescent probe (Solarbio) by FCM and CLSM.

### PD-L1 expression analysis

The membrane PD-L1 level and total PD-L1 expression in B16-F10 cells were evaluated by FCM and CLSM after being stained with immunofluorescence antibodies, respectively. In brief, B16-F10 cells were treated with various formulations for 24 h and collected for staining with APC-labeled PD-L1 antibody. Mean fluorescence intensity (MFI) was measured by FCM to quantify the relative level of membrane PD-L1 of B16-F10 cells after various treatments. Similarly, B16-F10 cells seeded onto confocal dishes overnight were fixed and permeabilized, and then stained with primary PD-L1 antibody, Alexa Fluor 488- labeled second antibody and DAPI in turn after the above treatments. The PD-L1 expressed within B16-F10 cells was imaged by CLSM. Furthermore, the quantitative analysis of total PD-L1 level in B16-F10 cells was analyzed by western blot (WB) assay as previously described [36].

### Animals and tumor models

C57BL/6 female mice (18 ~ 20 g) were purchased from Hubei Province Center for Disease Control and Prevention (Wuhan, China). All animal experiments were conducted following the Guidelines for Care and Use of Laboratory Animals and approved by the Animal Care and Use Committee of Huazhong University of Science and Technology. To establish the subcutaneous tumor model, B16-F10 cells resuspended in serum-free DMEM medium were subcutaneously inoculated to the right flank of the mice. When the tumor volumes grew to 50 mm^3^ or 200 ~ 300 mm^3^, these tumor-bearing mice were randomly sorted into groups for antitumor experiments in vivo and biodistribution study, respectively. To establish the tumor metastasis model, B16-F10 cells (1 × 10^5^) dispersed in PBS were intravenously injected into the mice. After 5 days, the treatments were first conducted to study the inhibition of lung metastasis in vivo.

### Pharmacokinetic profiles and distribution

The pharmacokinetic profiles and distribution of CPT after intravenous injection into the mice were analyzed according to previous reports [37, 38]. Briefly, free CPT and 2-BP/CPT-PLNs (5 mg/kg CPT-equivalent dose) were injected intravenously into the tumor-bearing or normal C57BL/6 female mice. At predesigned time points, the blood samples were withdrawn from the orbital vein of the above mice after administration and centrifuged to harvest plasma. Subsequently, the plasma samples were exacted with acidified acetonitrile to separate CPT and centrifuged to collect supernatant for further determination by a multi-mode microplate reader. After being administrated with CPT formulations for 24 h, the tumor-bearing mice were sacrificed and dissected to collect tumors and major organs. Then all tissue samples were weighed, homogenized, and centrifuged to collect supernatant for quantitative detection as above described.

### In vivo antitumor assay and histological analysis

B16-F10 tumor-bearing C57BL/6 female mice were randomly divided into five groups and intravenously injected with normal saline, free CPT, free 2-BP, CPT + 2-BP, and 2-BP/CPT-PLNs at an equivalent dose of 5 mg/kg CPT and 40 mg/kg 2-BP every two days for four times. The day of the first dosage was defined as Day 0, and the weights and tumor volumes of tumor-bearing mice were also recorded every two days. All mice were sacrificed and dissected to separate their tumors and major organs once the tumor volumes of partial mice exceeded 1500 mm^3^. The harvested tumors were weighted and imaged to evaluate the antitumor efficacy of 2-BP/CPT-PLNs i*n vivo*. Subsequently, the tumors and major organs were fixed with 4% PFA, paraffin-embedded, and sliced for hematoxylin and eosin (H&E) staining, Ki67 staining, or terminal deoxynucleotidyl transferase-mediated nick end labeling (TUNEL) assays. The blood biochemical analysis was conducted to further demonstrate the biosafety of 2-BP/CPT-PLNs during the above continuous and repeated administration.

The B16-F10 tumor metastasis model was treated with normal saline, free CPT, free 2-BP, CPT + 2-BP, and 2-BP/CPT-PLNs for 3 times every 3 days. All mice were sacrificed and dissected to separate their lungs and livers for analysis and visualization of metastasis nudes. To evaluate the systemic antitumor efficacy of 2-BP/CPT-PLNs, the marketed drugs including CPT-11 and αPD-L1 were introduced as a control to analyze the life survival of subcutaneous B16-F10 tumor-bearing mice after these treatments. Briefly, C57BL/6 mice were inoculated with B16-F10 cells (2 × 10^5^) for six days and then treated with normal saline, CPT-11, αPD-L1, CPT-11 + αPD-L1, and 2-BP/CPT-PLNs for 3 times every 3 days. The administrated doses of CPT-11 and αPD-L1 were 7.5 mg/kg and 100 µg per mouse, respectively. The injected dose of 2-BP/CPT-PLNs was CPT-equivalent of 5 mg/kg. The survival status of the mice was recorded daily. The mouse was euthanized and recorded as one dead item once its subcutaneous tumor exceeded 1500 mm^3^.

### In vivo antitumor immune response

To study the antitumor immune response induced by 2-BP/CPT-PLNs in vivo, the tumors and spleens were separated from the mice after the antitumor treatments as described previously. Then these tissues were cut into small pieces, digested, and dispersed into single-cell suspensions for further immunofluorescence staining. The harvested cells were stained with FITC anti-CD45, APC anti-PD-L1, APC anti-CD3, PE anti-CD4, FITC anti-CD8 or PE/Cy7 anti-Granzyme B in ice-cold PBS, and then immediately analyzed by FCM. Moreover, the tumor slides were immunofluorescence stained and then visualized by CLSM to evaluate CD8^+^ T cells infiltration and PD-L1 expression level in tumor sites. Immune-associated cytokines levels including IL-2, IL-6, IFN-γ, TNF-α, and TGF-β in tumors were also determined by the corresponding ELISA Test Kit (Solarbio).

### Statistical analysis

All experimental data were presented as means ± standard deviation (SD) following the statistical analysis to calculate the significant differences between two groups by a student’s t-test. Statistical significances were recorded as *p < 0.05, **p < 0.01, ***p < 0.001, and no significances were recorded as *N.S*.

## Results and discussion

### Rational design and characterization of 2-BP/CPT-PLNs

**Fig. 2:**
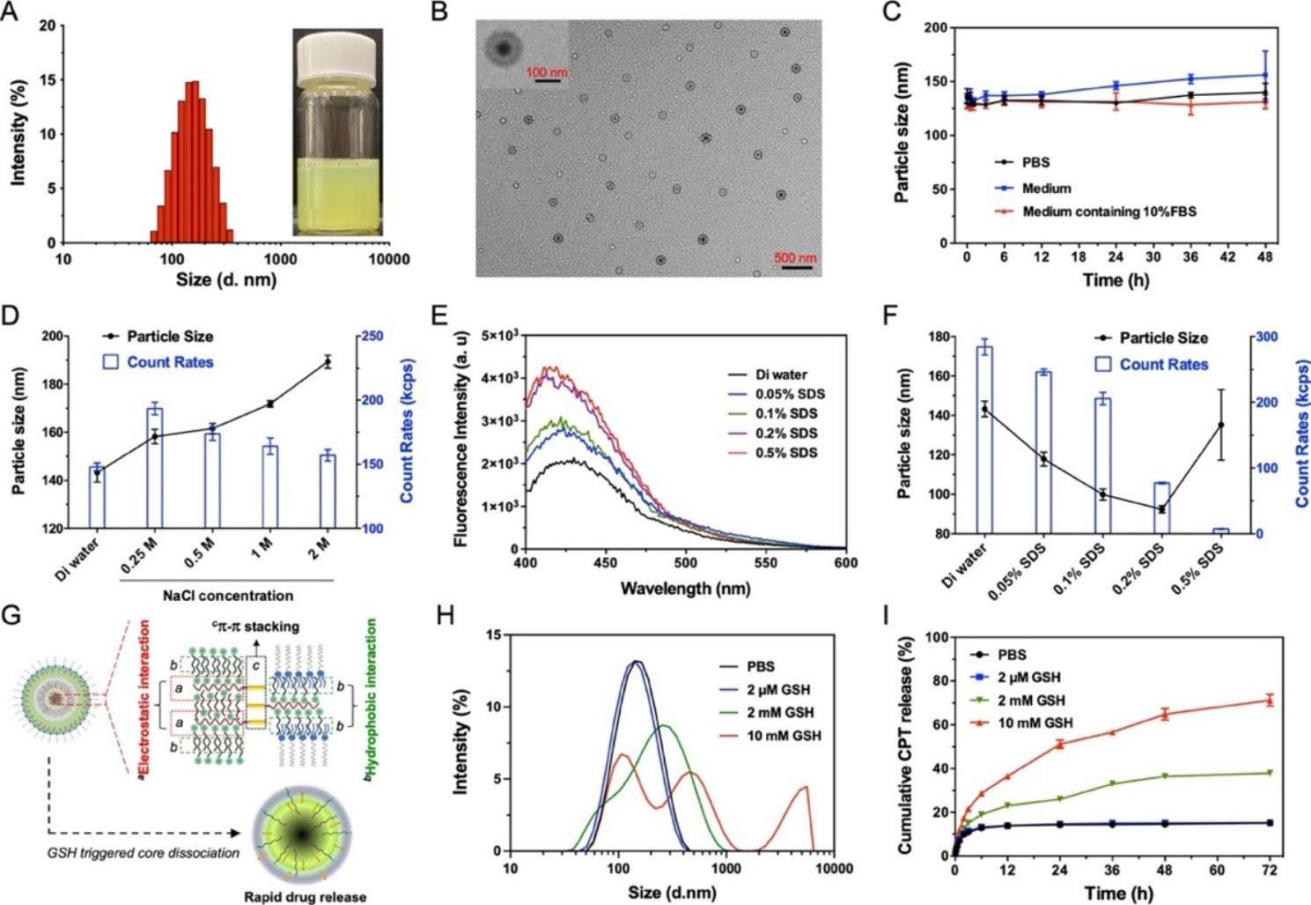
The characterizations and self-assembly behavior of 2-BP/CPT-PLNs. **A** Hydrodynamic size distribution and photograph of 2-BP/CPT-PLNs. The physical picture of the 2-BP/CPT-PLNs formulation was inserted. **B** TEM images of 2-BP/CPT-PLNs. **C** Particle size changes of 2-BP/CPT-PLNs in PBS, or medium in the presence or absence of FBS (n = 3). **D** Particle size and count rate changes of 2-BP/CPT-PLNs in various concentrations of NaCl solutions (n = 3). **E, F** Fluorescence spectra, particle size, and count rate changes of 2-BP/CPT-PLNs in aqueous solutions of SDS (n = 3). **G** Schematic illustration of self-assembly driven forces and GSH-triggered drug release of 2-BP/CPT-PLNs. **H** Particle size distribution and cumulative CPT release profiles of 2-BP/CPT-PLNs in PBS (10 mM, pH 7.4) solutions in the absence or presence of GSH (2 µM, 2 mM, 10 mM) (n = 3). Data were represented as mean ± SD

Considering the potential toxicity caused by introducing organic solvents and simplification of quality management in preparation, polymer-lipid hybrid nanoparticles were usually manufactured with solvent evaporation and ultrasonic emulsification technology [32, 39]. GSH-responsive cationic polymer-prodrug CPT-ss-PAEEP_10_ was successfully synthesized and characterized by ^1^H-NMR (Additional file 1: Fig. S1). Herein, 2-BP/CPT-PLNs were fabricated by the co-assembly of 2-BP, CPT-ss-PAEEP_10_, and DSPE-PEG at the rational feed ratio with the convenient evaporation-emulsification method (Fig. 1A). As expected, the introduction of CPT-ss-PAEEP_10_ significantly reversed the highly negative potentials of nano constructions (2-BP-PLNs) assembled from 2-BP and DSPE-PEG and improved the assembly efficiency of 2-BP and particle size uniformity of the hybrid nanoparticles (Additional file 1: Table S2). The optimized feed ratio of 1:8:1 (DSPE-PEG:2-BP:CPT-ss-PAEEP_10_) was selected to prepare 2-BP/CPT-PLNs because of their high encapsulation efficacy (~ 99%). 2-BP/CPT-PLNs exhibited a uniform multilayer core-shell spherical structure with an average hydrodynamic size of 142.2 nm and a slightly positive surface potential of nearly 12 mV in an aqueous solution (Fig. 2A, B). It was acceptable for the further in vivo application that 2-BP/CPT-PLNs showed approximately 5% CPT loading efficiency. As we expected, the introduction of CPT-ss-PAEEP_10_ as a cationic helper-polymer further improved the stability of 2-BP/CPT-PLNs under physiological conditions in the presence of DSPE-PEG (Fig. 2C). The driving force for the self-assembly of 2-BP/CPT-PLNs was studied by fluorescence spectra and DLS technology. 2-BP/CPT-PLNs showed significantly increased particle size and slightly decreased count rates following the gradually increased amount of additive NaCl (Fig. 2D), owning to the limited disruption of electrostatic interactions and excellent resistance to the high concentration of salt ions. Conversely, SDS as a strong surfactant remarkably dissociated the constructure of 2-BP/CPT-PLNs causing the considerable enhancement of fluorescent signals and sharply decreased particle size and count rates (Fig. 2E, F). As illustrated in Fig. 2G, the electrostatic interactions, hydrophobic interactions, and π-π stacking played a vital role in driving stable co-assembly of the three components to form 2-BP/CPT-PLNs. Furthermore, GSH concentration-dependent increased fluorescent signals ensured that intracellular high-level GSH could trigger the core dissociation of 2-BP/CPT-PLNs to boost drug release after their entrance into tumor cells (Fig. 2H, I; Additional file 1: Fig. S2).

### 2-BP/CPT-PLNs enabled synergistic chemotherapy from 2-BP and CPT in vitro

**Fig. 3:**
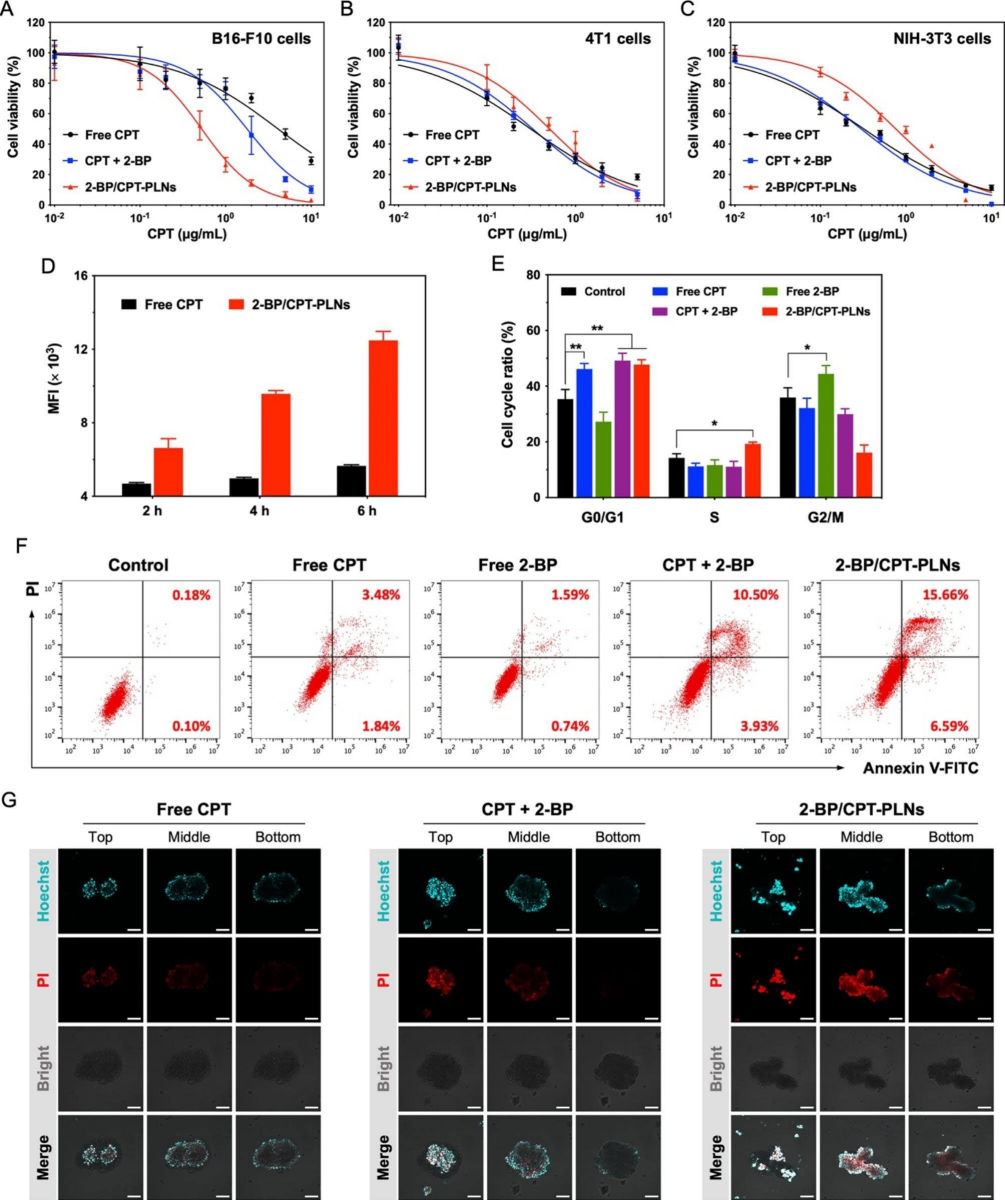
In vitro antitumor effects of 2-BP/CPT-PLNs. **A-C **In vitro cytotoxicity of 2-BP/CPT-PLNs against B16-F10 cells, 4T1 cells, and NIH-3T3 cells (n = 6). **D** Mean fluorescence intensity (MFI) was measured by flow cytometry (FCM) to evaluate cellular uptake of 2-BP/CPT-PLNs by B16-F10 cells (n = 3). **E, F** Cell cycle arrest and cell apoptosis induced by 2-BP/CPT-PLNs were analyzed by FCM (n = 3). **G** Cell apoptosis within B16-F10 multicellular spheroids induced by 2-BP/CPT-PLNs was evaluated by CLSM. The Bar: 100 μm. Data were represented as mean ± SD. *p < 0.05 or **p < 0.01 represented significance

To verify the down-regulation of PD-L1 induced by 2-BP for targeted PD-L1 degradation and potential cytotoxicity of 2-BP/CPT-PLNs in various cell lines, B16-F10 melanoma and 4T1 breast cancer cells were screened for PD-L1 expression and chemosensitization induced by 2-BP. As shown in Fig. 3A-C, 2-BP/CPT-PLNs exhibited stronger cytotoxicity than free CPT against B16-F10 cells rather than 4T1 cells and NIH-3T3 cells. Especially, the addition of 2-BP caused significant CPT chemosensitization and PD-L1 downregulation in B16-F10 cells (Additional file 1: Fig. S3, Table S3). Actually, the inherent cytotoxicity of 2-BP against various cells was dose-dependent and serum concentration-dependent (Additional file 1: Fig. S4). The cytotoxic dose of 2-BP was matched to the relative dose from 2-BP/CPT-PLNs against B16-F10 cells but not for 4T1 cells. Thence, the B16-F10 cell line was selected as a tumor model for antitumor evaluation in vitro and in vivo own to its higher intracellular GSH level, membrane PD-L1 level, and sensitivity to 2-BP for synergistic chemotherapy [40, 41]. As we expected, 2-BP/CPT-PLNs significantly enhanced intracellular CPT accumulation in B10-F10 cells in contrast to free CPT (Fig. 3D; Additional file 1: Fig. S5). We further investigated the in vitro antitumor mechanism to reveal that the remarkable cell cycle arrest in the G0/G1 and S phase, and greatly improved cell apoptosis induced by 2-BP/CPT-PLNs, might play a vital role in inhibiting tumor cells growth or directly killing tumor cells (Fig. 3E, F; Additional file 1: Fig. S6). Moreover, as shown in Fig. 3G, 2-BP/CPT-PLNs caused the apparent disintegration of B16-F10 multicellular tumor spheroids (MCTs) and significantly increased cell apoptosis within MCTs compared with free CPT or the combination of CPT and 2-BP (Additional file 1: Fig. S7). Herein, it was reasonably speculated that 2-BP/CPT-PLNs would enable the synergistic chemotherapeutic effect of 2-BP and CPT to facilitate further antitumor immunotherapy in vivo.

### 2-BP/CPT-PLNs induced efficient ICD and PD-L1 downregulation in vitro

**Fig. 4:**
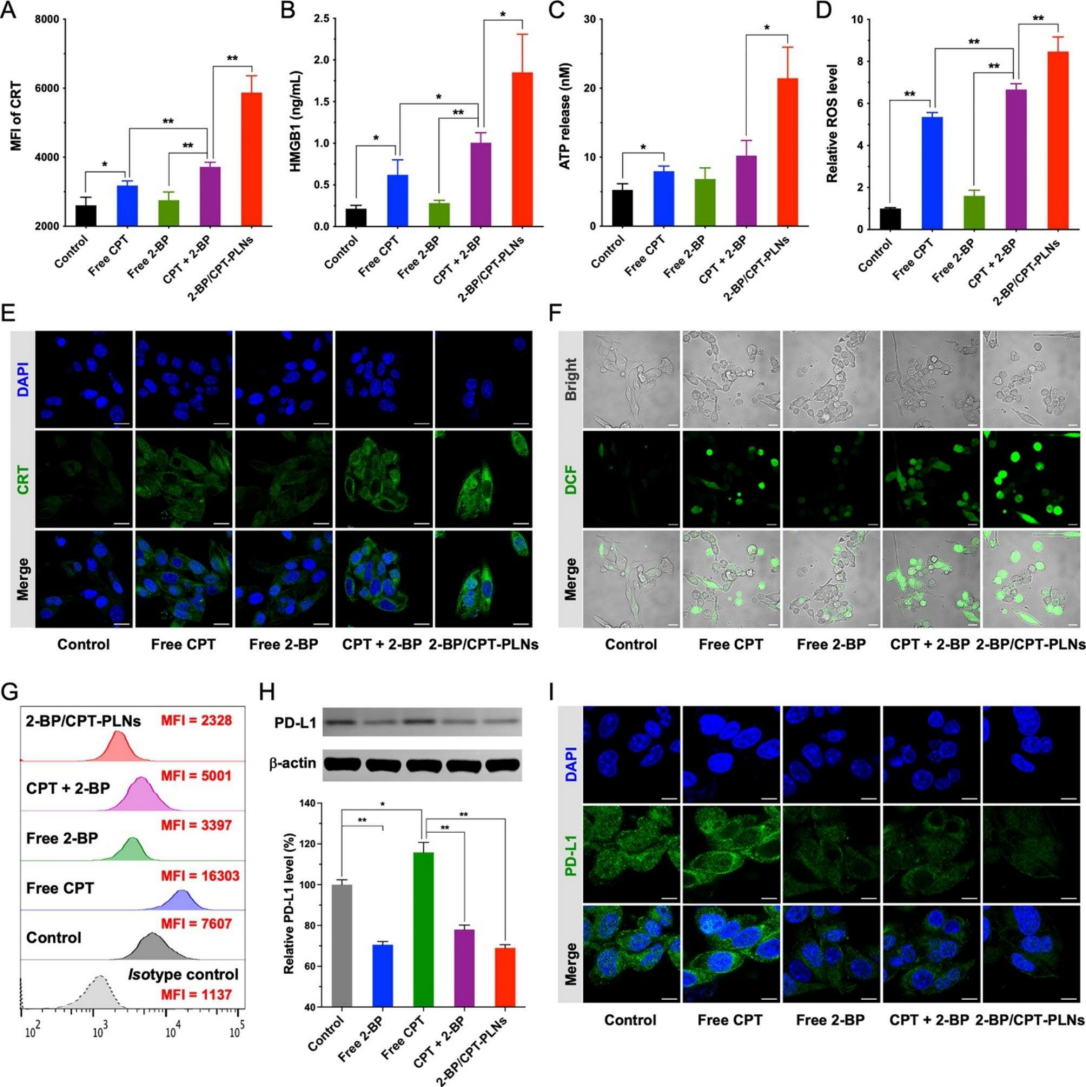
The ability evaluation of ICD induced by 2-BP/CPT-PLNs. **A-D** CRT exposure, HMGB1, ATP release, and upregulation of intracellular ROS level induced by 2-BP/CPT-PLNs against B16-F10 cells were analyzed by FCM, and measured by microplates after being treated with the corresponding ELISA test kits (n = 3). **E** CRT expression of B16-F10 cells after chemotherapy was evaluated by CLSM. Bar: 20 μm. **F** The intracellular ROS level of B16-F10 cells after being stained with DCFH-DA was evaluated by CLSM. Bar: 50 μm. **G** The down-regulation of membrane PD-L1 level in B16-F10 cells induced by 2-BP/CPT-PLNs. **H** Total PD-L1 protein levels of B16-F10 cells after various treatments were evaluated by WB assay (n = 3). **I** Immunofluorescence staining of PD-L1 in B16-F10 cells with various treatments. Bar: 10 μm. Data were represented as mean ± SD. *p < 0.05 or **p < 0.01 represented significance

The synergistic antitumor effect of chemo-immunotherapy benefits from the activation of immune response induced by ICD and ICB [42]. Herein, we further investigated whether 2-BP/CPT-PLNs would induce efficient ICD and downregulate PD-L1 for sustained ICB. 2-BP/CPT-PLNs had the potential to induce apoptosis and even directly kill cancer cells as previously illustrated, thus inducing CRT translocation to the outer cell membrane and triggering HMGB1 and ATP release to the extracellular matrix as typical ICD responses (Fig. 4A-C, E). Apart from cell apoptosis, ROS production involved with endoplasmic reticulum (ER) stress induced by 2-BP/CPT-PLNs might have a heavy influence on ICD (Fig. 4D, F) [43]. As we expected, 2-BP/CPT-PLNs induced stronger ICD than free CPT, free 2-BP, and their combination. Furthermore, it was noteworthy that 2-BP/CPT-PLNs would significantly decrease PD-L1 expression level on the membrane of B16-F10 cells as well as the intracellular PD-L1 storage compared with the two groups of control and free CPT (Fig. 4G-I). It was accepted that the efficient intracellular 2-BP delivery via positive-charged hybrid nanoparticles and boosted 2-BP release triggered by GSH, might play a vital role in promoting the PD-L1 degradation induced by its destabilization without palmitoylation to offset PD-L1 adaptive upregulation induced by CPT chemotherapy.

### Synergistic antitumor chemo-immunotherapy by 2-BP/CPT-PLNs in vivo

Before exploring the antitumor efficacy of 2-BP/CPT-PLNs in vivo, we first investigated the pharmacokinetic profiles and distribution of CPT to ensure efficient drug delivery to tumor lesion sites via these polymer-lipids hybrid nanoparticles (Additional file 1: Fig. S8). It was supposed that 2-BP/CPT-PLNs remarkably delayed CPT clearance in blood and enhanced CPT accumulation by 2.7-folds in tumor sites compared with free CPT. It could be interpreted as the combined achievements from long circulation induced by PEGylation, the potentially enhanced accumulation of nanoparticles by enhanced permeability and retention (EPR), and the promoted intracellular delivery by cellular uptake rather than passive diffusion [38, 44].

**Fig. 5:**
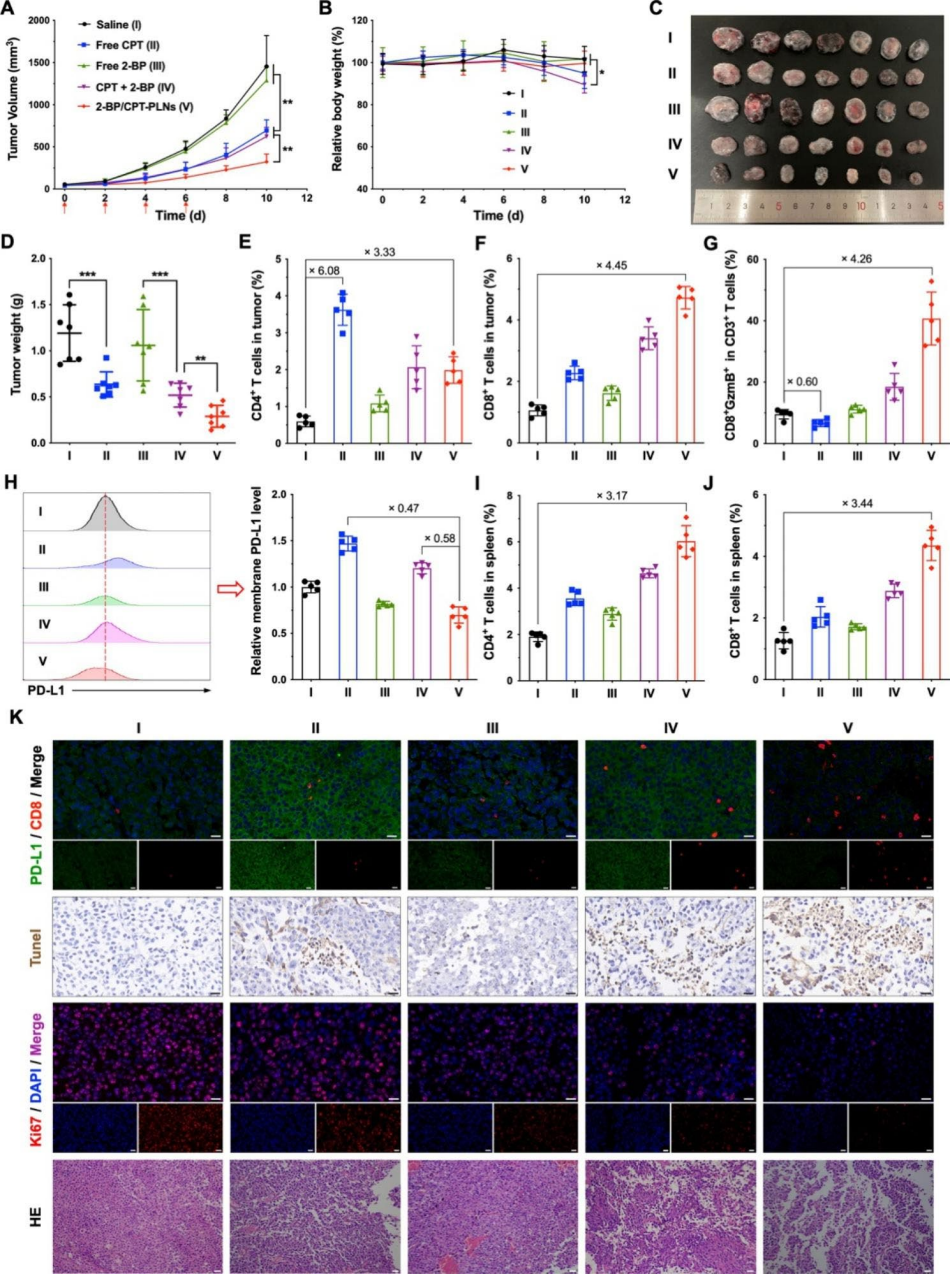
Tumor growth inhibition and immune response induced by chemo-immunotherapy with 2-BP/CPT-PLNs in vivo. **A, B** Tumor growth files and body weight changes of B16-F10 tumor-bearing mice during the chemo-immunotherapy. The red arrows represented the time points of every intravenous administration. **C, D** The morphology and weights of excised tumors from mice after the first treatments for 10 days. **E-G** Quantification analysis of CD4^+^, CD8^+^, and CD8^+^GzmB^+^ T cells (gated from CD3^+^ T cells) infiltration within tumors. **H** Relative membrane PD-L1 expression level of tumor cells (gated from CD45^**-**^ cells) within tumor tissues. **I-J** Quantification analysis of CD4^+^, CD8^+^ T cells in spleens from the above-administrated mice. **K** H&E staining and immunofluorescence staining of Ki67, Tunel, PD-L1, and CD8 of tumor slides. Bar: 50 μm. Data were represented as mean ± SD (n = 7 in **A-D**, and n = 5 in **E-J**). *p < 0.05, **p < 0.01 or ***p < 0.001 represented significance

Next, the subcutaneous B16-F10 melanoma model was established to evaluate the tumor growth inhibition and antitumor immune response of 2-BP/CPT-PLNs *in vivo.* As shown in Fig. 5A, 2-BP/CPT-PLNs exhibited excellent tumor growth inhibition in contrast to free CPT. It was reasonably speculated that the combination of CPT and 2-BP (CPT + 2-BP) hardly enhanced the tumor inhibitory rate compared with CPT, owing to the deficiency of 2-BP access to tumor sites and entrance into tumor cells caused by its poor water solubility and fast clearance. In addition, the addition of 2-BP might exacerbate the side effect of free CPT and further cause significant weight loss in mice after the above treatments (Fig. 5B). As above mentioned, the intravenous administration with free 2-BP almost did not influence tumor growth even as a complementary treatment of free CPT (Fig. 5C, D). To further check the potential activation of antitumor immune response induced by 2-BP/CPT-PLNs, all tumors and spleens were separated from the experimental tumor-bearing mice after administration with various formulations for the subpopulation ratio analysis of T cells or PD-L1 level on the membrane of tumor cells by FCM (Fig. 5E-J; Additional file 1: Fig. S9). Indeed, 2-BP/CPT-PLNs induced 3.33-folds of CD4^+^ T cells and 4.45-folds of CD8^+^ T cells infiltration in tumors higher than the saline group. Furthermore, 2-BP/CPT-PLNs could activate systemic antitumor immune response characterized by both promoted frequencies of CD4^+^ T cells and CD8^+^ T cells over 3 times within spleens. Herein, 2-BP/CPT-PLNs enabled the downregulation of PD-L1 level on the tumor cell surface to offset the potential PD-L1 adaptive upregulation during chemotherapy and the remediation of the immunosuppressive microenvironment by regulating multiple cytokines including interleukin-2 (IL-2), interleukin-6 (IL-6), interferon- γ (IFN- γ), tumor necrosis factor-α (TNF-α) and transforming growth factor-β (TGF-β) for regulation of innate immunity against pathogens (Fig. 5H; Additional file 1: Fig. S10). Therefore, the promotion of the CD8^+^ T cells infiltration and cytotoxic killing ability against tumor cells within tumor lesion locations was a reason for significantly strengthened tumor cell apoptosis induction and proliferation inhibition by chemo-immunotherapy with 2-BP/CPT-PLNs (Fig. 5K). We also noticed that 2-BP/CPT-PLNs could prevent the emergence of tiny tumor metastasis nodules within livers or lungs (Additional file 1: Fig. S11). As our previous speculation, the combination of free CPT and free 2-BP (CPT + 2-BP) caused the severe disorder of blood biochemical indicators including ALT, AST, BUN, CR, and LDH which might throw light on the above weight loss after being treated with their combined application (Additional file 1: Fig. S12).

### Melanoma metastasis inhibition and systemic antitumor efficacy by chemo-immunotherapy

**Fig. 6:**
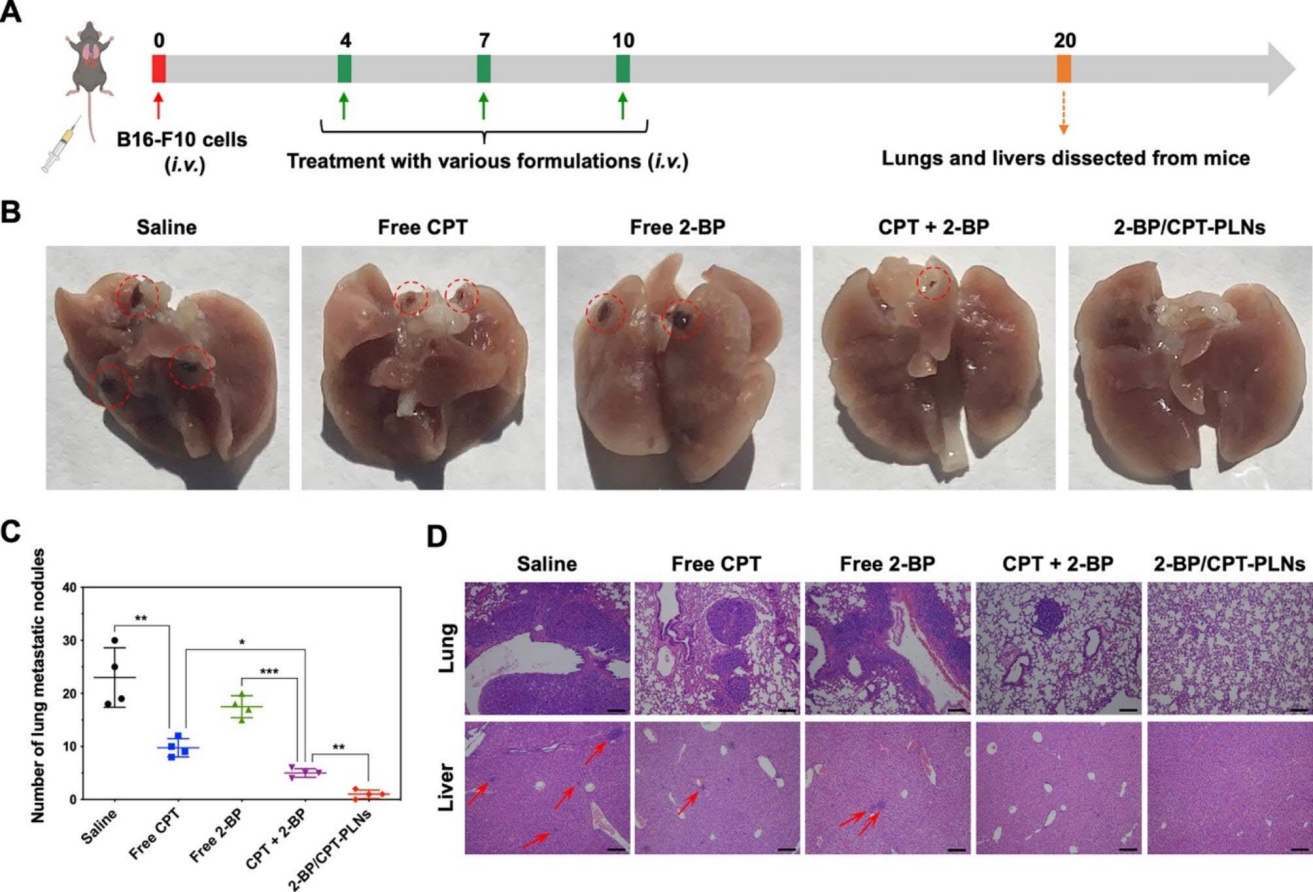
2-BP/CPT-PLNs prevented B16-F10 melanoma metastasis in vivo. **A** Schedule and timeline for constructing the B16-F10 tumor metastasis model and dissecting lungs and livers from mice after treatments with various formulations. **B** The images of the excised lungs. The red dotted circles represented melanoma metastasis nudes within the lungs. **C** The digital analysis of lung metastasis nudes (n = 4). **D** H&E staining of lung and liver slides. The red arrows represented the tiny metastasis nudes within livers. Bar: 100 μm. *p < 0.05, **p < 0.01 or ***p < 0.001 represented significance

**Fig. 7:**
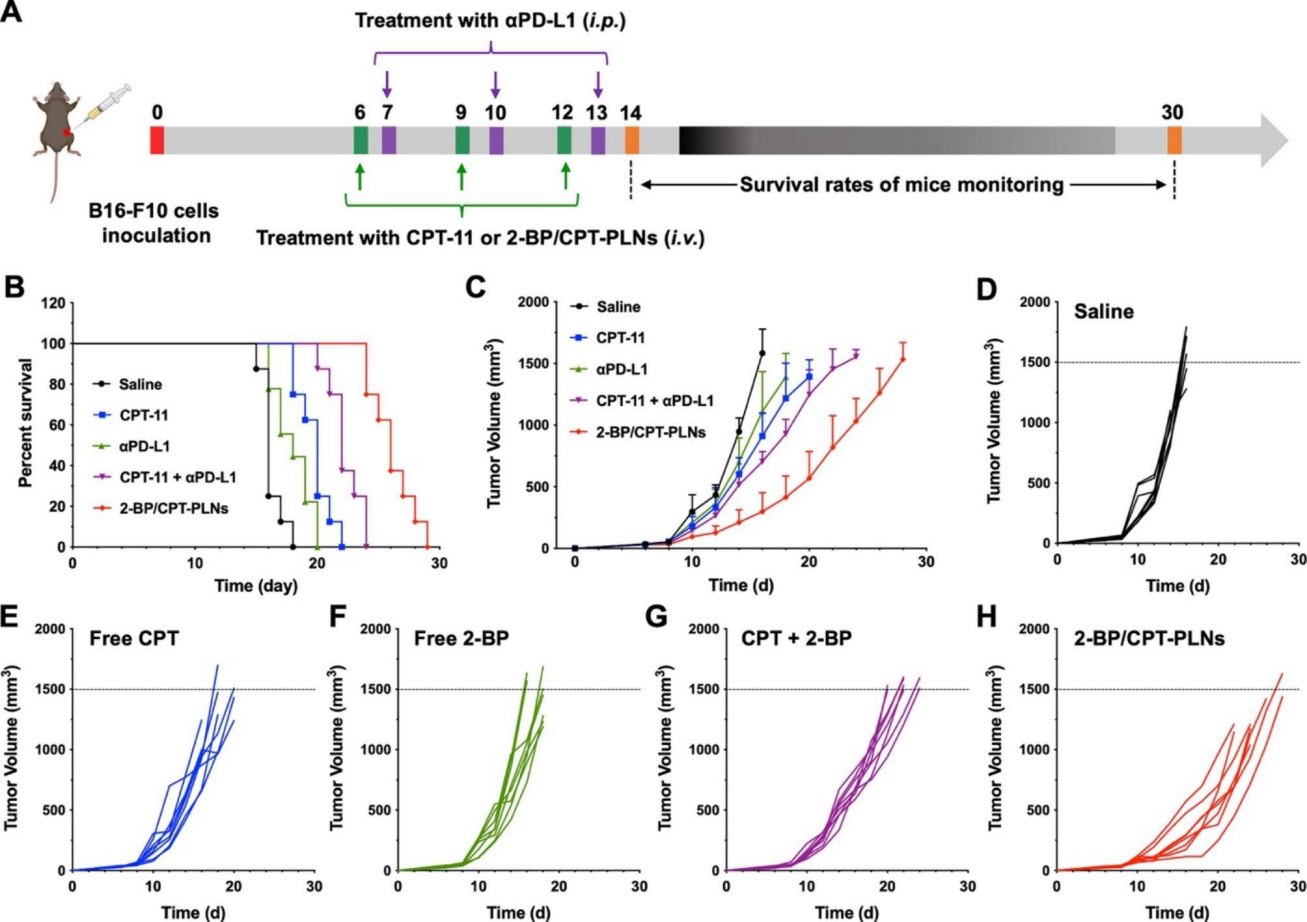
2-BP/CPT-PLNs improved systemic antitumor efficacy and prolonged the survival of B16-F10 tumor-bearing mice. **A** Schematic illustration of the treatment regimen for the subcutaneous B16-F10 tumor model. **B-H** Tumor growth files and survival files of tumor-bearing mice after the above programmatic treatments (n = 8). Data were represented as mean ± SD

To explore the capacity of 2-BP/CPT-PLNs to prevent tumor metastasis, we developed the melanoma metastasis model in vivo via intravenous injection with B16-F10 cells. As illustrated in Fig. 6A, the lungs and livers were dissected from C57BL/6 mice that underwent exogenous melanoma invasion and therapeutic trials intravenously. Inevitably, 2-BP/CPT-PLNs significantly inhibited the lung metastasis of melanoma and prevented its potential metastasis to the liver completely (Fig. 6B-D). By contrast, free drugs with the existence of molecular forms including CPT, and 2-BP, and their combination might be limited by their fast clearance in blood without access to the invasive tumor cells, thus causing their inefficient anti-metastasis against melanoma in vivo. The above results were consistent with the inhibition effects of orthotopic tumor metastasis to lungs or livers in previous tumor growth inhibition assays. Considering the severe aggressiveness of melanoma, we further confirmed the systemic antitumor efficacy of 2-BP/CPT-PLNs to reduce lesion size and prolong life survival by chemo-immunotherapy proved previously. Herein, we introduced the marketed drugs including CPT-11 and PD-L1 monoclonal antibody (αPD-L1) widely applicated for antitumor treatments in the clinic, as control treatments to evaluate the systemic therapeutic efficacy against B16-F10 melanoma model (Fig. 7A). Unsurprisingly, 2-BP/CPT-PLNs prevent melanoma progression and significantly prolonged the life survival of tumor-bearing mice compared with the combination of CPT-11 and αPD-L1 (CPT + αPD-L1) (Fig. 7B-D). Given that the traditional chemo-immunotherapy was achieved by the combined administration in completely different ways, 2-BP/CPT-PLNs enabled the synchronized drug delivery to tumor lesion sites for better synergistic chemo-immunotherapy.

## Conclusion

In conclusion, we developed novel polymer-lipid prodrug-based hybrid nanoparticles to achieve excellent antitumor synergistic chemo-immunotherapy beyond the conventional combination of CPT-11 and αPD-L1 via sensitizing CPT chemotherapy, activating the antitumor immune response, and improving antitumor immune killing capacity of cytotoxic T cells against melanoma. Especially, 2-BP as a fatty acid derivative acted a double role in fabricating the novel polymer-lipid nanoparticles (PLNs) for chemo-immunotherapy. GSH-responsive and biodegradable polymeric prodrug CPT-ss-PAEEP_10_ as a cationic helper polymer, could help to stabilize PLNs co-assembled with 2-BP and DSPE-PEG and facilitate site-specific delivery of two water-insoluble bioactive components in vivo. PD-L1 degradation by inhibiting palmitoylation with such a cheap lipid derivative to replace monoclonal antibodies for ICB, would provide a highly valuable paradigm for manufacturing novel lipid-based nanoparticles via lipid metabolism intervention for onotherapy.

## Electronic supplementary material

Below is the link to the electronic supplementary material.

Supplementary Material 1
