# Supplementary material for: Bioactive fatty acid analog-derived hybrid nanoparticles confer antibody-independent chemo-immunotherapy against carcinoma
Source: J Nanobiotechnology. 2023 Jun 8;21:183. doi: 10.1186/s12951-023-01950-y (PMC10249277; doi:10.1186/s12951-023-01950-y)
Supplement: Supplementary file 1 — Supplementary Material 1 [file 12951_2023_1950_MOESM1_ESM.docx]

**Supporting Information**

**Bioactive Fatty Acid Analog-derived Hybrid Nanoparticles Confer Antibody-independent Chemo-immunotherapy against Carcinoma**

Xi Tan^1^, Chenhui Wang^2^, Hong Zhou^1^, Shuting Zhang^1^, Xuhan Liu^3^, Xiangliang Yang^1,4^, and Wei Liu^1,4^*

^1^ College of Life Science and Technology, Huazhong University of Science and Technology, Wuhan 430074, P.R. China

^2^ The Key Laboratory for Human Disease Gene Study of Sichuan Province and the Department of Laboratory Medicine, Sichuan Provincial People’s Hospital, University of Electronic Science and Technology of China, Chengdu 611731, P.R. China

^3^ Department of Emergency Medicine, Shenzhen University General Hospital, Shenzhen University Clinical Medical Academy, Shenzhen 518060, P.R. China

^4^ National Engineering Research Center for Nanomedicine, Huazhong University of Science and Technology, Wuhan 430074, P.R. China

*Correspondence: Wei Liu, wliu@hust.edu.cn

**
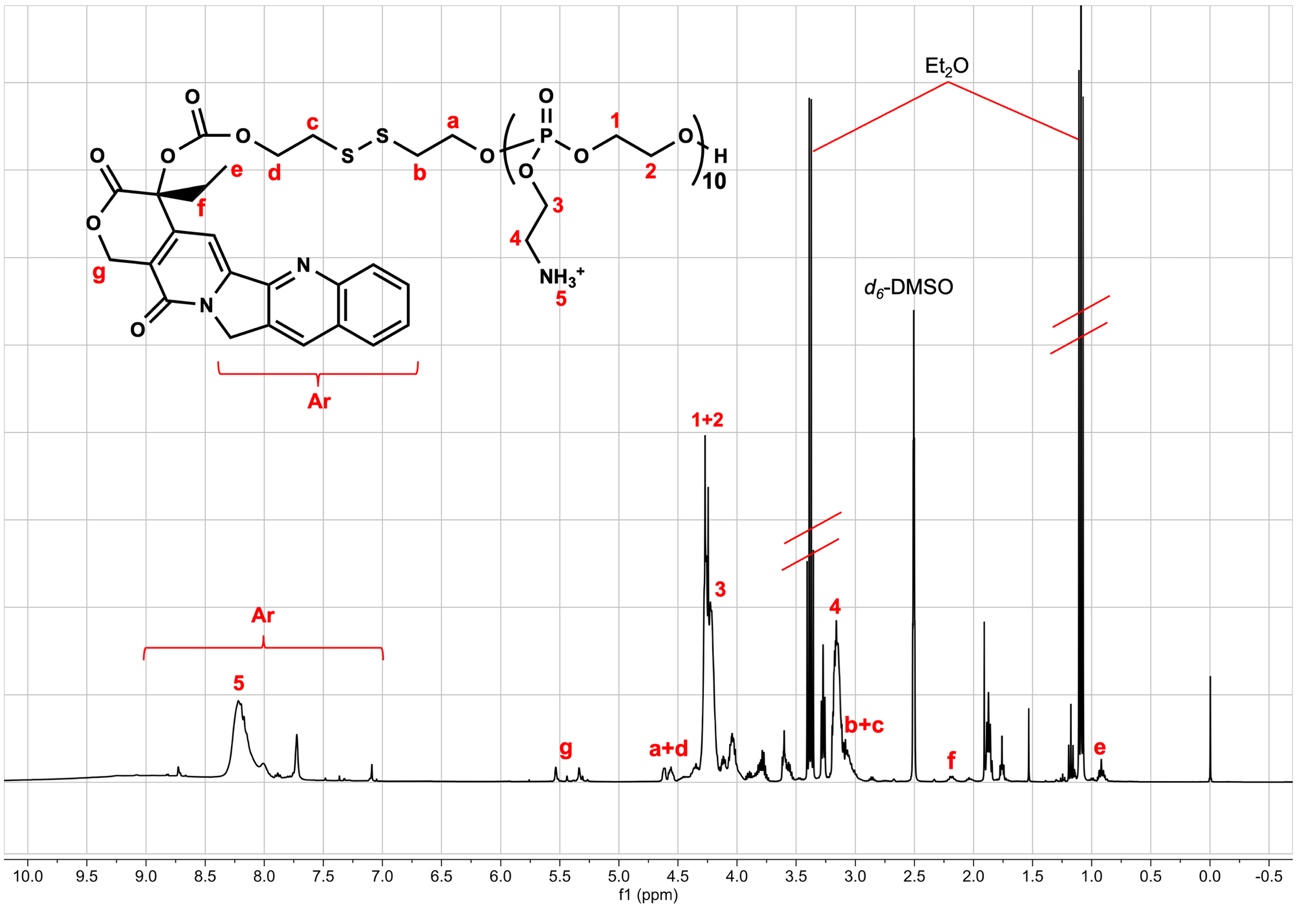
**

**Fig. S1** The ^1^H NMR (400 MHz) spectra of CPT-ss-PAEEP_10_ in *d_6_*-DMSO (ppm). The degree of polymerization (DP=10) was calculated according to the ratio of the characteristic peak integral area (*A_1-3_*/*Ae*).

**Fig. S2** Fluorescence spectra of 2-BP/CPT-PLNs after incubation with various concentrations of GSH for 3 h.

**
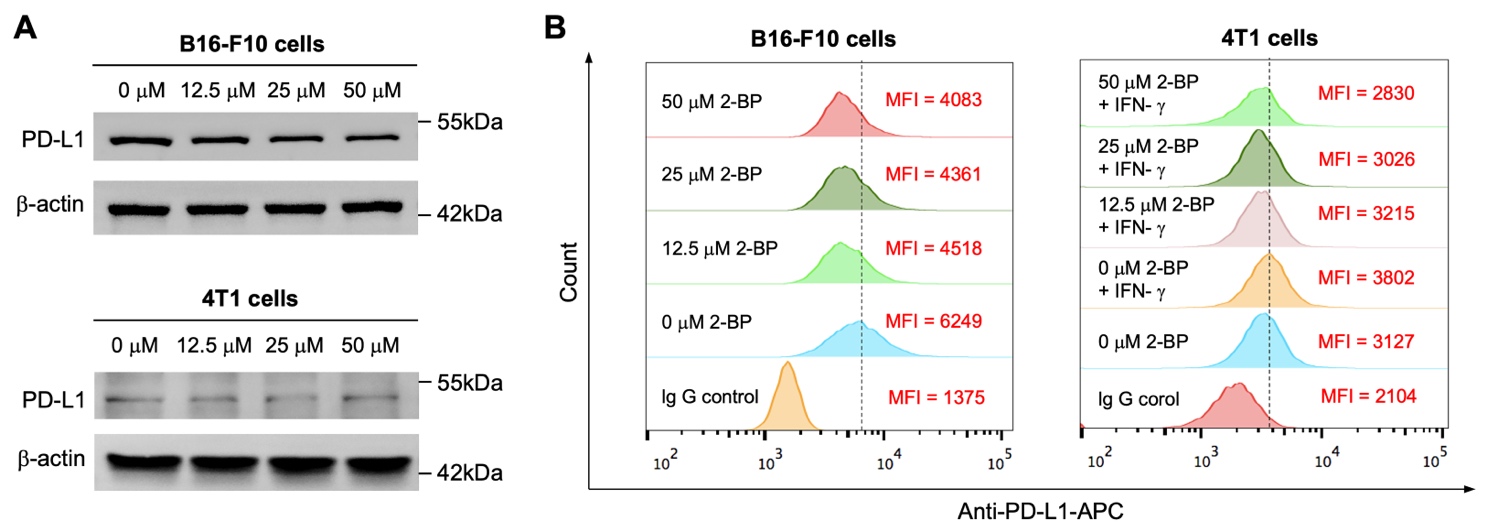
**

**Fig. S3** The concentration-dependent PD-L1 downregulation induced by 2-BP. **A** The western blot (WB) assay results of PD-L1 in B16-F10 cells or 4T1 cells after being treated with various concentrations of 2-BP for 24 h. **B** The PD-L1 expression levels on the membrane of B16-F10 cells or 4T1 cells after the above treatments with various concentrations were analyzed by FCM. Here, IFN-γ was used to upregulate the PD-L1 expression level on the membrane of 4T1 cells.


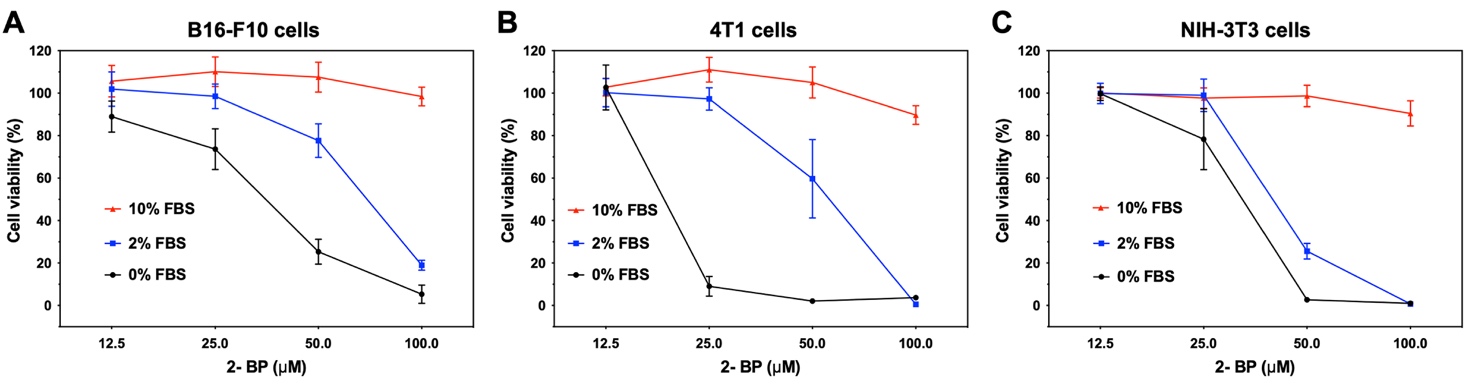


**Fig. S4** The serum concentration-dependent cytotoxicity of 2-BP *in vitro*. **A-C** The cytotoxicity of 2-BP against various cells in presence of fetal bovine serum (FBS) was evaluated by MTT assay. Data were represented as mean ± SD (n=6).


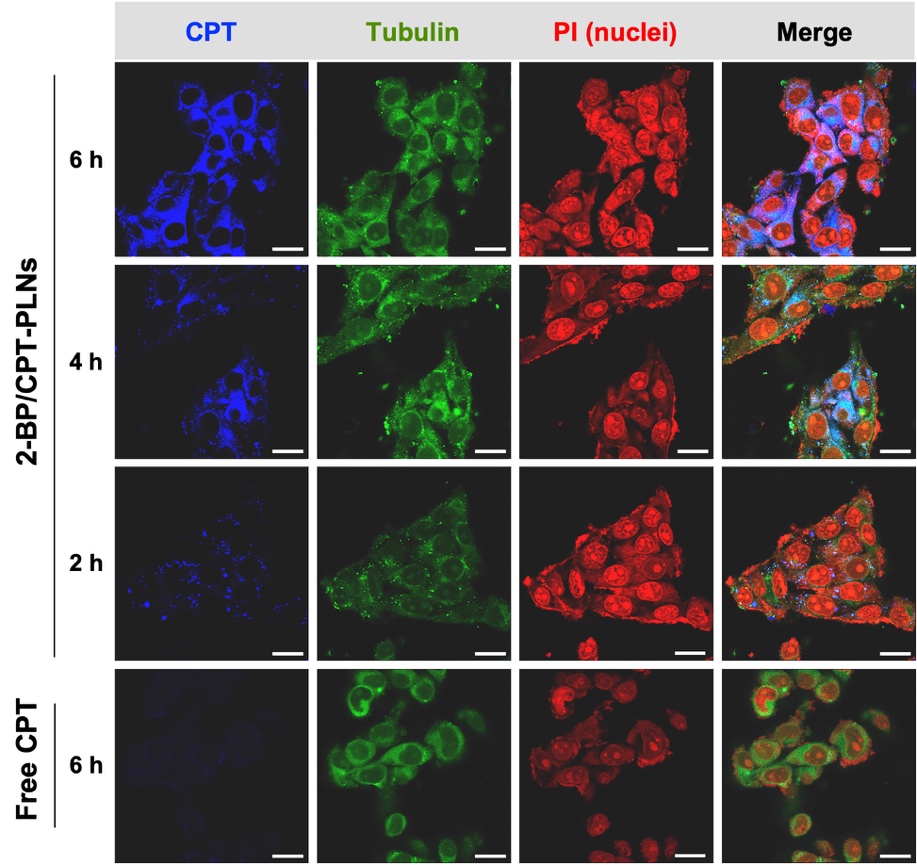


**Fig. S5** The intracellular traffic of CPT delivered by 2-BP/CPT-PLNs was observed by confocal laser scanning microscopy (CLSM). Bar: 20 μm.

**
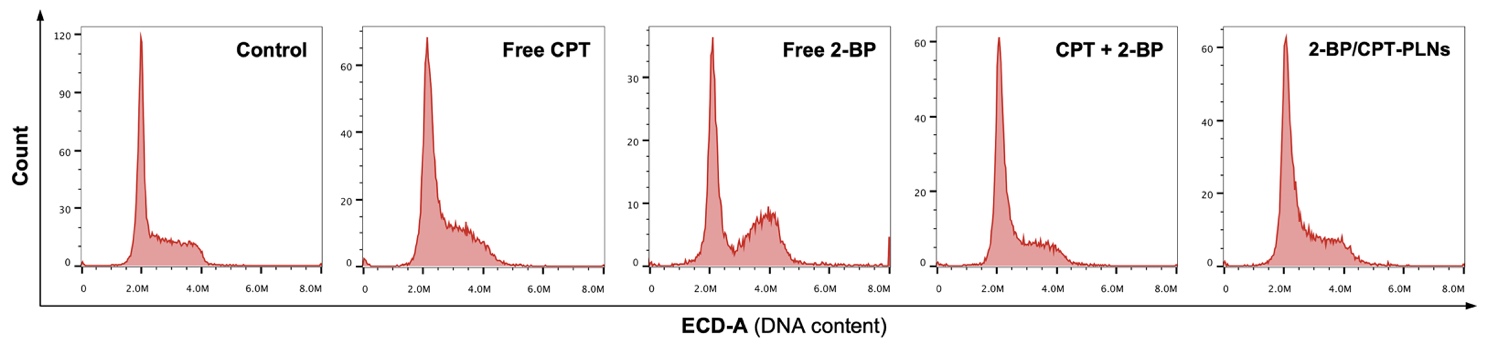
**

**Fig. S6** The cell cycle arrest analysis of B16-F10 cells after being treated with various formulations was conducted with FCM.


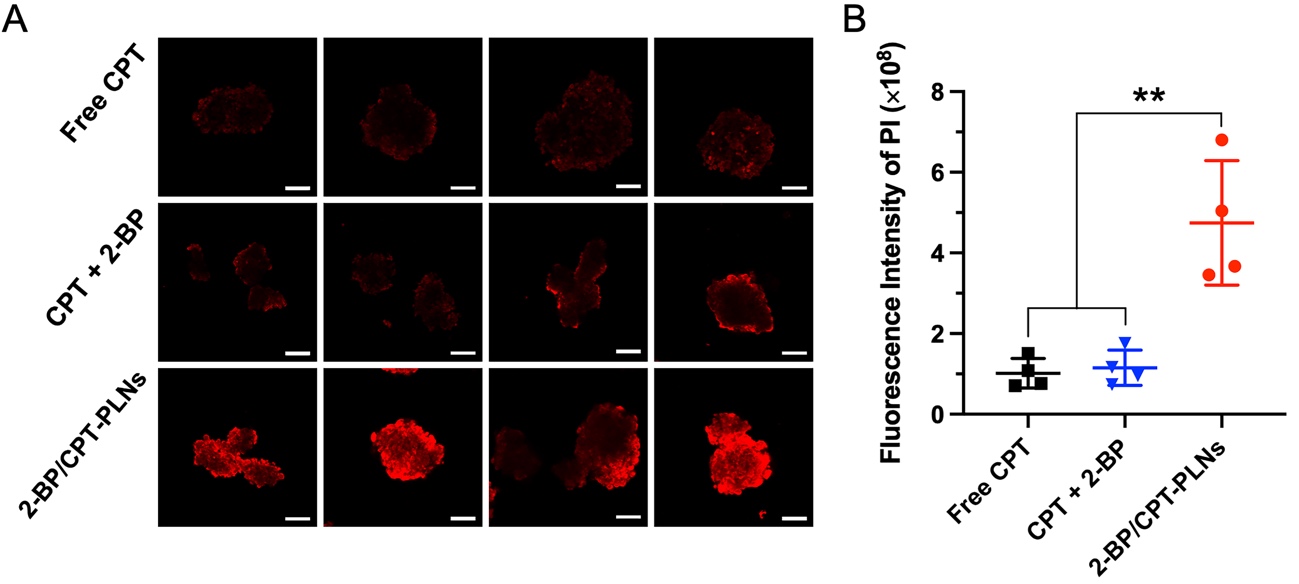


**Fig. S7** Cell apoptosis analysis of B16-F10 multicellular spheroids induced by 2-BP/CPT-PLNs. **A** B16-F10 cells pretreated with various formulations were stained with PI and imaged by CLSM. The Bar: 100 μm. **B** The fluorescence intensities of PI within B16-F10 multicellular spheroids. Data were represented as mean ± SD. ***p* < 0.01 represented signiﬁcance.

**
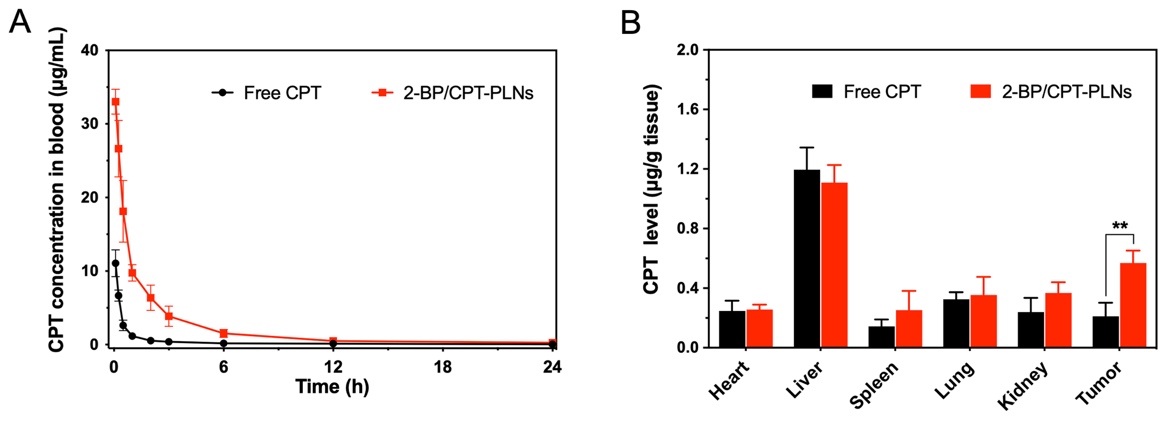
**

**Fig. S8** The pharmacokinetic profiles and distribution of 2-BP/CPT-PLNs *in vivo*. **A** The CPT concentration in blood was analyzed at predesigned time points after intravenous administration with free CPT or 2-BP/CPT-PLNs into mice. **B** The amount of CPT in tumor or other organs of B16-F10 tumor-bearing mice were measured after intravenous administration with free CPT or 2-BP/CPT-PLNs for 24 h. Data were represented as mean ± SD (n = 4).


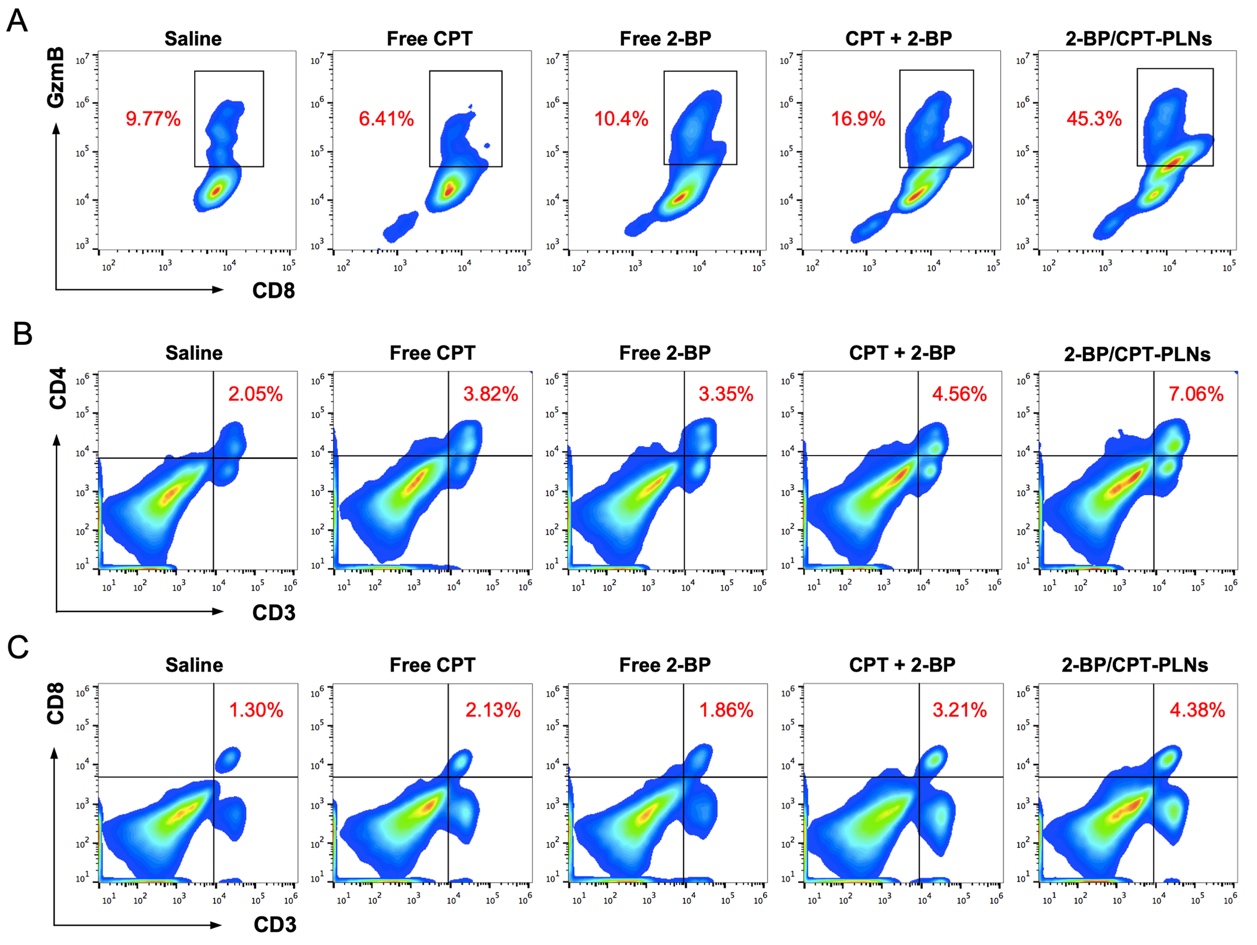


**Fig. S9** The subpopulation ratio analysis of T cells within tumors or spleen by FCM. **A** The subpopulation ratio of CD8^+^GzmB^+^ T cells in CD3^+^ T cells within tumors. **B** The subpopulation ratio of CD3^+^CD4^+^ T cells and CD3^+^CD8^+^ T cells within spleens.


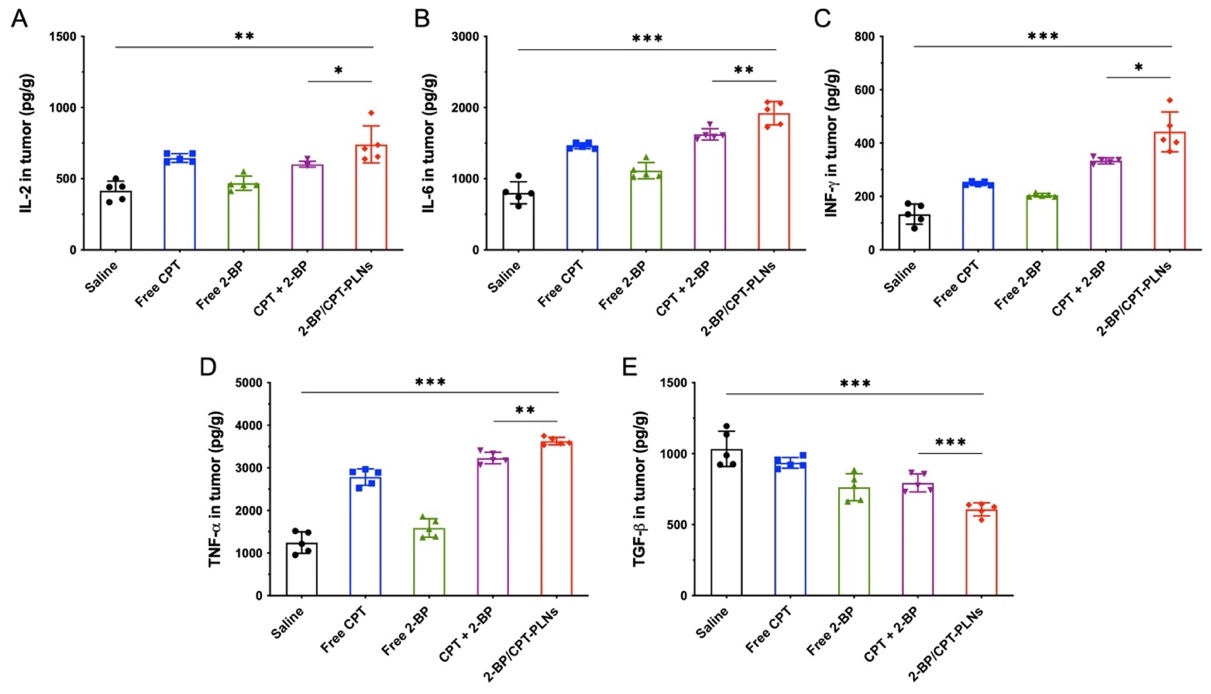


**Fig. S10** **A-E** The expression level of intratumoral immune-related cytokines including IL-2, IL-6, IFN-γ, TNF-α and TGF-β was measured by ELISA. Data were represented as mean ± SD (n = 5). **p* < 0.05, ***p* < 0.01, ****p* < 0.001.

**
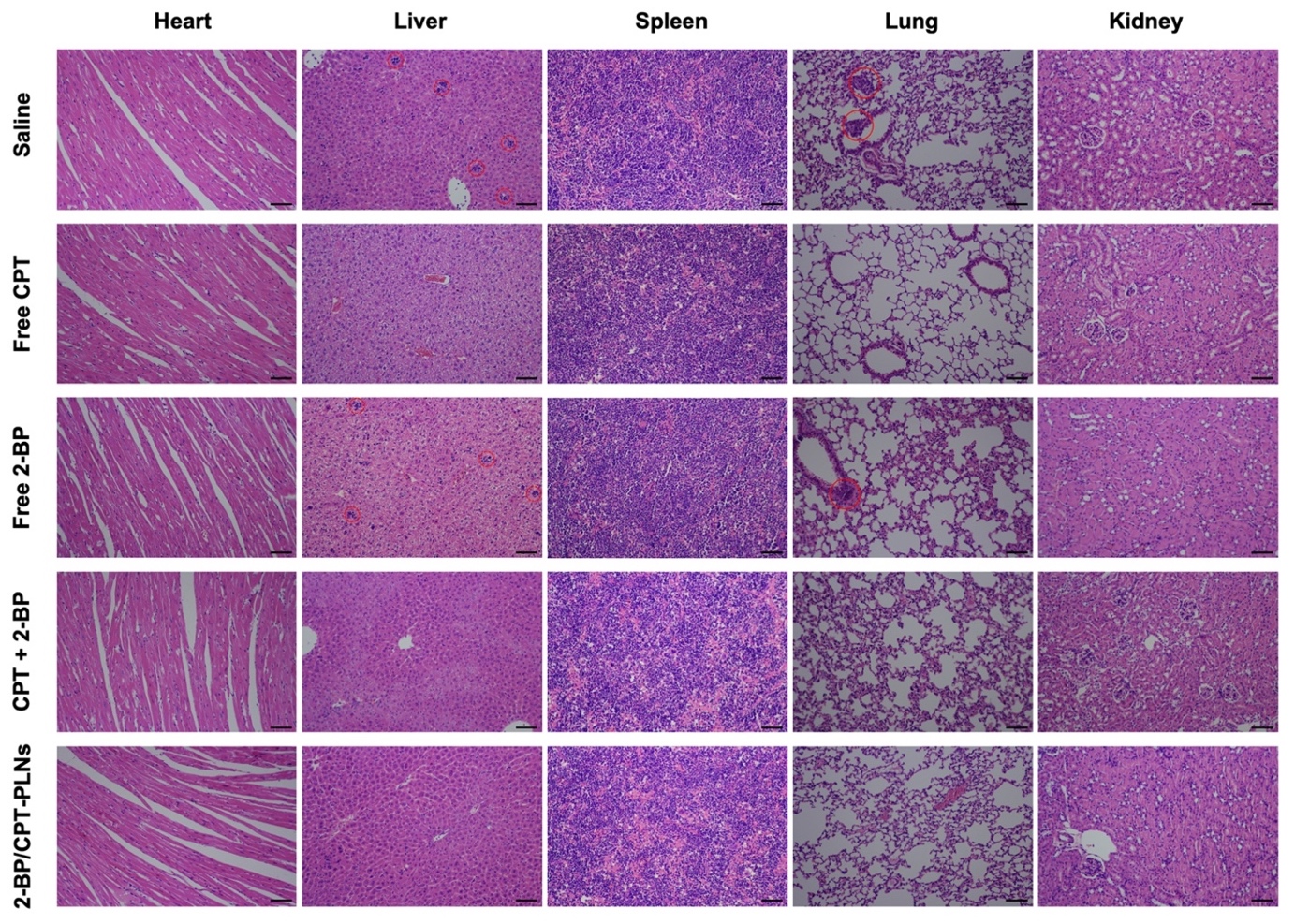
**

**Fig. S11** The H&E staining of hearts, livers, spleens, lungs, and kidneys excised from the B16-F10 tumor-bearing mice after treatments with various formulations (as illustrated in Fig. 5A). The red circles represented the melanoma metastasis nudes within livers or lungs. Bar: 50 μm.

**Fig. S12** The analysis of blood biochemical indicators including ALT, AST, BUN, CR, LDH, and CK-MB in collected blood from B16-F10 tumor-bearing mice after being treated with various formulations on Day 10 as illustrated in Fig. 5A).

**Table S1** Antibodies used for flow cytometry (FC) or IHC/IF in this study.

| **Antibody** | **Company** | **Catalog No.** | **Application** | **Dilution** |
| --- | --- | --- | --- | --- |
| APC anti-mouse PD-L1 | Biolegend | 124312 | FC | 1:100 |
| APC anti-Rat IgG2b,κ | Biolegend | 400612 | FC | 1:100 |
| FITC anti-mouse CD45 | Biolegend | 103108 | FC | 1:200 |
| APC anti-mouse CD3ε | Biolegend | 100312 | FC | 1:200 |
| PE anti-mouse CD4 | Biolegend | 100511 | FC | 1:200 |
| FITC anti-mouse CD8a | Biolegend | 100705 | FC | 1:200 |
| PE/Cy7 anti-human/mouse Granzyme B | Biolegend | 372214 | FC | 1:20 |
| Recombinant anti-PD-L1 antibody | Abcam | ab213480 | IF | 1:125 |
| CRT Polyclonal antibody | Proteintech | 10292-1-AP | FC, IF | 1:100 |
| Alexa Fluor 488 goat anti-rabbit IgG (H+L) | Beyotime | A0428 | FC, IF | 1:200 |

**Table S2** The characterizations of various polymer-lipid hybrid nanoparticles assembled from DSPE-PEG and 2-BP with or without CPT-ss-PAEEP_10_ at different feed ratios.

| **Formulations** | **Molar ratios**  **(DSPE-PEG/2-BP/CPT-ss-PAEEP_10_)** | **Particle size (nm)** | **PDI** | **Zeta potential (mv)** |
| --- | --- | --- | --- | --- |
| **2-BP-PLNs** | 1 : 1 : 0 | 159.8 ± 1.6 | 0.249 ± 0.021 | -37.7 ± 1.5 |
|  | 1 : 2 : 0 | 298.8 ± 7.3 | 0.307 ± 0.042 | -46.6 ± 0.6 |
|  | 1 : 4 : 0 | 228.6 ± 1.5 | 0.421 ± 0.013 | -47.3 ± 0.8 |
|  | 1 : 8 : 0 | Precipitation | — | — |
| **2-BP/CPT-PLNs** | 1 : 8 : 1 | 142.2 ± 2.3 | 0.202 ± 0.023 | 12.1 ± 0.6 |

**Table S3** The half-inhibitory CPT concentration (IC_50_, μg/mL) of various formulations against multiple cell lines.

| **Formulations** | **B16-F10 cells** | **4T1 cells** | **NIH-3T3 cells** |
| --- | --- | --- | --- |
| Free CPT | 4.15 ± 0.82 | 0.31 ± 0.05 | 0.35 ± 0.03 |
| CPT + 2-BP | 1.79 ± 0.26 | 0.33 ± 0.03 | 0.30 ± 0.03 |
| 2-BP/CPT-PLNs | 0.52 ± 0.05 | 0.51 ± 0.07 | 0.76 ± 0.10 |
